# Left-sided Appendicitis with Intestinal Non-rotation: A Case Report

**DOI:** 10.31729/jnma.7274

**Published:** 2022-04-30

**Authors:** Ashish Mohan Bhattarai, Yogendra Devkota, Ayush Mohan Bhattarai

**Affiliations:** 1 Department of Radiodiagnostics, Nobel Medical College, Biratnagar, Morang, Nepal; 2 Department of Surgery, Nobel Medical College, Biratnagar, Morang, Nepal; 3 Department of Medicine, Nepalese Army Institute of Health Sciences, Sanobharyang, Kathmandu, Nepal

**Keywords:** *appendicitis*, *case reports*, *peritonitis*

## Abstract

Appendicitis rarely presents with an left lower abdominal pain especially when the intestine is non-rotated or malrotated. Its diagnosis becomes quite troublesome to clinicians and delays prompt intervention. Non-rotation is the most common type of intestinal malrotation. Here, we present a case of a 40-year-old female with previously undiagnosed intestinal non-rotation with left lower abdominal pain and features of localised peritonitis. Abdominal ultrasonography and multidetector computerised tomography showed left-sided appendicitis with intestinal non-rotation. Diagnostic laparoscopy followed by explorative laparotomy and appendectomy was performed. Clinicians and surgeons are usually trained to diagnose and operate on right-sided appendix, thus, diagnosing and promptly intervening on left-sided appendicitis is quite challenging. Left-sided appendicitis must be kept in mind if a patient presents with left lower abdominal pain. Timely radiological scans like ultrasonography and computerised tomography scans help in prompt diagnosis in these cases.

## INTRODUCTION

Acute appendicitis is the most common surgical cause of acute abdomen in the Emergency Room (ER).^1^ Leftsided appendicitis with intestinal non-rotation adds more confusion for prompt diagnosis and surgical intervention due to abnormal clinical presentation. It may be clinically confused with diverticulitis for which Ultrasonography (USG) and Computed Tomography (CT) scans are very useful for diagnosing the dilemmatic cases.^2^ Intestinal malrotation refers to the congenital abnormal position of small and large bowel loops inside the peritoneal cavity. Non-rotation is the most common type of intestinal malrotation with small bowels on the right side and large bowels on the left side of the abdomen.^3^

## CASE REPORT

A 40-year-old female with no known comorbidities presented to our emergency department with complaints of progressive, constant, left lower abdominal pain for two days. The pain was associated with nausea, vomiting, and increased body temperature. The patient explained that pain initially was confined to the periumbilical region which later on shifted to the left lower quadrant. There was no history of vomiting out of blood, abdominal distention, and passage of blood mixed stool. She is a non-smoker and non-alcoholic. She had a prior history of repair of umbilical hernia seven years back. On examination, she was noted to be febrile (101°F), with a blood pressure of 120/70 mm Hg and a pulse rate of 84 beats per minute. Abdominal examination revealed a soft abdomen with left lower quadrant guarding, rebound tenderness, and normal per-rectal examination.

**Figure 1:**
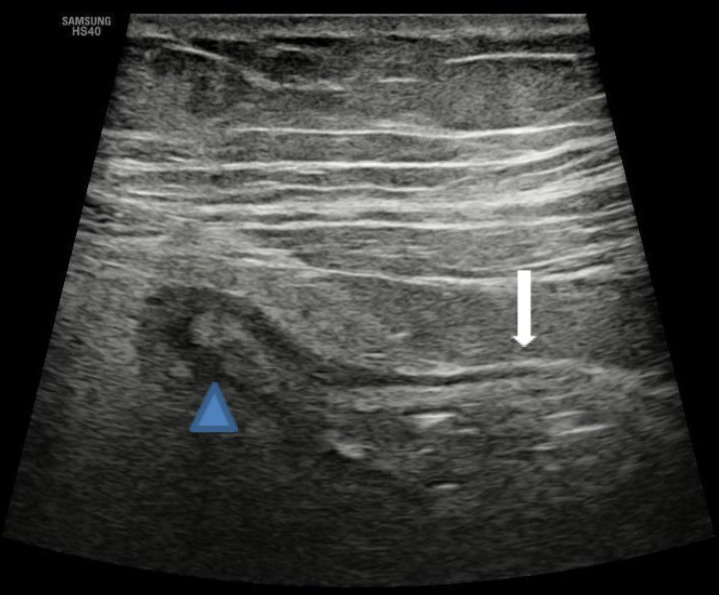
Ultrasonography of the abdomen shows a blind-ended, aperistaltic, non-compressible, tubular structure (block arrow) with surrounding inflammatory changes arising from the caecum (arrowhead).

With a presumptive diagnosis of diverticulitis, abdominal ultrasonography was done which revealed a blind-ended, aperistaltic, non-compressible, tubular structure with surrounding inflammatory changes arising from the left iliac fossa, and CT was advised to confirm the possibility of left-sided appendicitis (Figure 1).

**Figure 2:**
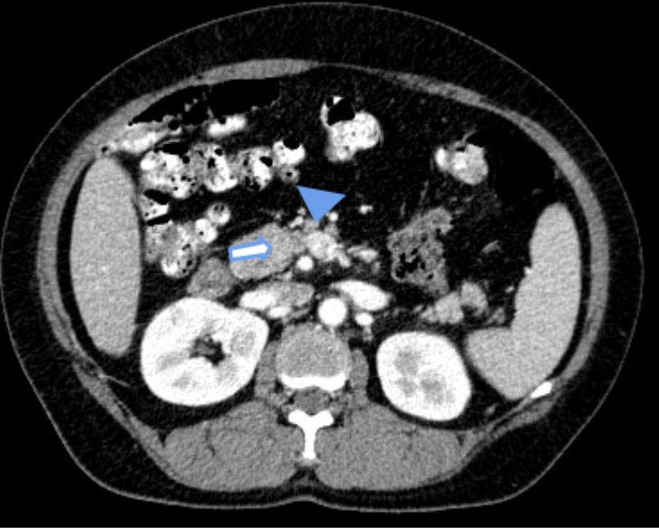
Contrast enhanced CT scan of the abdomen shows SMA (block arrow) lying on the right side of SMV (arrowhead) revealing an inverse relationship between them.

An inverse relationship between Superior Mesenteric Vein (SMV) and Superior Mesenteric Artery (SMA) was noted, with the artery to the right of the vein (Figure 2).

**Figure 3:**
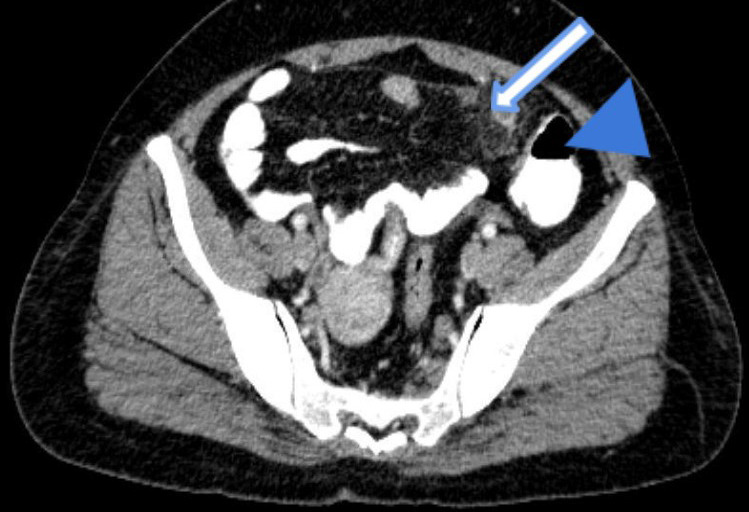
Axial image of contrast-enhanced CT scan of abdomen and pelvis shows inflamed appendix (block arrow) arising from the caecum (arrowhead) in the left iliac fossa.

A plain CT scan followed by oral and intravenous contrast was done, which showed a left-sided inflamed appendix of 10 mm in diameter with surrounding peri appendiceal fat stranding (Figure 3).

**Figure 4:**
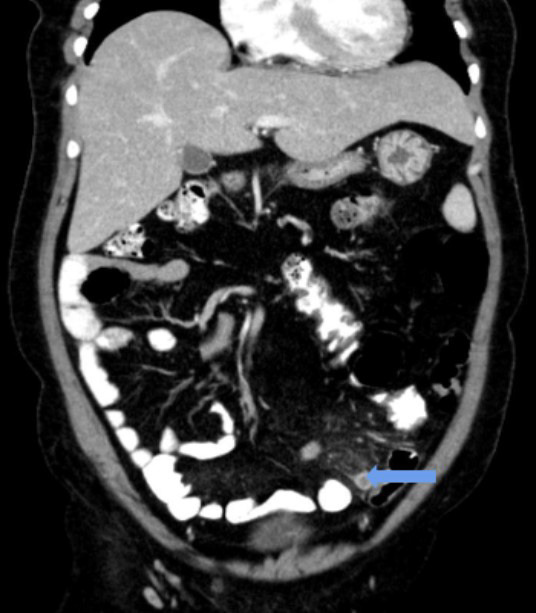
Coronal image of contrast-enhanced CT scan of the abdomen shows left-sided appendicitis (block arrow) with peri-appendiceal fat stranding.

Intestinal non-rotation with caecum and large bowel on the left side and small bowel on the right side of the abdomen was noted (Figure 4).

**Figure 5:**
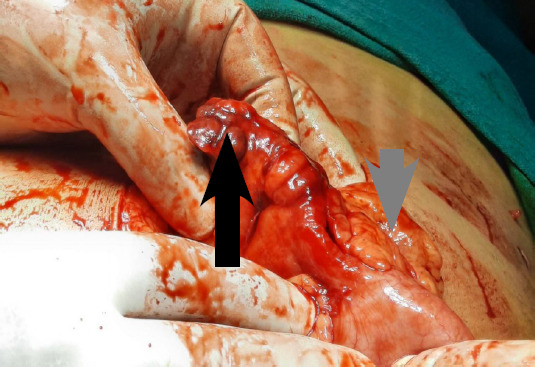
Intraoperative findings show an inflamed appendix (black arrow) and caecum (grey arrow) arising from the left iliac fossa.

Thus, the diagnosis of left acute appendicitis with intestinal non-rotation was made. Diagnostic laparoscopy was performed which revealed early lump formation and due to minimal surgeon's experience in relatively different anatomy, a lower midline incision was made. Intraoperatively, the appendix was found to be inflamed with a healthy base attached to the caecum in the left iliac fossa with minimal periappendiceal fat stranding (Figure 5).

Appendectomy was done and closed in layers. The postoperative period was uneventful and the patient was discharged on the fifth postoperative day with pharmacological medications. She was on regular follow-up and was made a good recovery. The histology of the specimen showed an inflamed appendix.

## DISCUSSION

Left-sided acute appendicitis can be seen in association with two different congenital anomalies: intestinal malrotation and situs inversus. Intestinal malrotation occurs congenitally either due to non-rotation or an incomplete rotation of the primitive gut around the axis of the superior mesenteric artery.^4,5^ It occurs due to failure of normal 270° anticlockwise rotation of the midgut. A study described there are various subtypes of intestinal malrotation, non-rotation being the commonest and mostly asymptomatic.6 In intestinal non-rotation (Stringer type Ia), there is no rotation of the duodenum and the large bowel after 90°, therefore, the small bowel lies on the right side and the large bowel including the caecum and appendix lies on the left side of the abdomen.^6^

Acute appendicitis presents with a variety of symptoms, typically presenting with migrating periumbilical pain towards the right iliac fossa associated with nausea, vomiting, and high-grade fever. The classical presentation is only seen in 50% of the cases and in addition, those patients presenting with left lower abdominal pain are easily misdiagnosed causing a high chance of a missed or delayed diagnosis of left-sided appendicitis.^7^ Initially, our patient was also diagnosed with diverticulitis due to atypical clinical presentation, intestinal non-rotation being the culprit. Left-sided appendicitis with intestinal non-rotation (Stringer type Ia) was diagnosed only after ultrasonography and a CT scan of the abdomen were done.^6^ Due to delayed diagnosis and features of early lump formation in diagnostic laparoscopy, our patient had to undergo explorative laparotomy. Though left-sided appendicitis associated with intestinal malrotations is very rare, it is uncommon. Various cases of appendicitis with an atypical location similar to our case have been described.^5,8-10^

In conclusion, diagnosing left-sided appendicitis is quite challenging for clinicians and surgeons leading to delayed intervention. It must be also kept in mind that appendicitis presents with a wide range of clinical signs and symptoms and left-sided appendicitis is one of the differentials of left lower abdominal pain.
